# An integrated single-cell transcriptomic and microbiome dataset of orbital adipose tissue in thyroid eye disease

**DOI:** 10.1038/s41597-026-08064-8

**Published:** 2026-08-13

**Authors:** Xiang Xu, Fei Wang, Weiqi Liu, Haowen Li, Yingyu Wang, Yuan Li, Bing Wang, Mingming Liu, Yang Li

**Affiliations:** 1 Institute of Microcirculation, Chinese Academy of Medical Sciences & Peking Union Medical College, Beijing, 100005 China; 2 International Center of Microvascular Medicine, Chinese Academy of Medical Sciences, Beijing, 100005 China; 3 Beijing Tongren Eye Center, Beijing Key Laboratory of Intraocular Tumor Diagnosis and Treatment, Beijing Tongren Hospital, Capital Medical University, Beijing, 100730 China; 4 Department of Ophthalmology, Air Force Medical Center, Beijing, 100142 China; 5 Diabetes Research Center, Chinese Academy of Medical Sciences, Beijing, 100005 China; 6 Beijing Institute of Ophthalmology, Beijing Tongren Hospital, Beijing Ophthalmology and Visual Science Key Lab, Capital Medical University, Beijing, 100730 China

## Abstract

Thyroid eye disease (TED), also historically referred to as thyroid-associated ophthalmopathy (TAO) or Graves’ orbitopathy (GO), is an autoimmune ophthalmopathy characterized by inflammation and adipogenesis within orbital adipose (OA) tissue. While the contribution of immune cell infiltration and fibroblast activation is recognized, the cellular heterogeneity and the potential role of local microbial dysbiosis in the orbital microenvironment remain poorly defined. Here, we present a multi-omics dataset derived from OA of patients with TED and healthy controls collected at Beijing Tongren Hospital. The dataset comprises single-cell RNA sequencing (scRNA-seq) data generated from human orbital adipose tissue and subcutaneous fat. Following quality control filtering and doublet removal, a total of 61,302 high-quality singlet cells were retained for downstream analyses, capturing the transcriptomic landscape of orbital fibroblasts, immune subsets, and vascular cells. In parallel, 16S rRNA gene sequencing was performed on orbital adipose tissue samples collected from the integrated study cohort, alongside environmental samples for contamination assessment, to characterize the low-biomass orbital microbiome DNA signatures. Raw sequencing reads, quality-controlled gene-expression matrices, and contamination-corrected microbial feature tables are provided. Our dataset provides an opportunity to investigate the crosstalk between host cellular immunity and potential local microbiome signatures, facilitating hypothesis generation regarding host-microbiome crosstalk and the exploration of novel therapeutic targets for TED.

## Background & Summary

Thyroid eye disease (TED)^1^, also historically referred to as thyroid-associated ophthalmopathy (TAO) or Graves’ orbitopathy (GO), represents the most common extrathyroidal manifestation of Graves’ disease^2,3^. The pathology is driven by an autoimmune response targeting the orbital connective tissue, leading to orbital fibroblast proliferation, adipogenesis, and hyaluronan accumulation^4^. Despite recent advances, the precise mechanisms initiating this local autoimmune cascade remain elusive^5^. Current research suggests a gut-eye axis^6,7^ where intestinal dysbiosis modulates immune responses^8^, however, the existence of locally resident microbial DNA in orbital tissue and its potential functional relevance remains a subject of intense debate and high clinical interest.

OA is distinct from other fat depots due to its neuroectodermal origin and complex cellular composition^9^. To date, an atlas integrating high-resolution cellular landscape with the local microbial environment of OA is lacking^10^. Understanding the microenvironment is important, as the interaction between resident fibroblasts and infiltrating T cells may be modulated by microbial antigens or metabolites^11^, potentially perpetuating chronic inflammation.

We established a cohort of TED patients undergoing orbital decompression and healthy controls undergoing cosmetic blepharoplasty at Beijing Tongren Hospital. We employed an experimental design to generate a parallel dataset of scRNA-seq (10x Genomics) and 16S rRNA gene sequencing (Illumina NovaSeq). Recognizing the low-biomass nature of orbital tissue^12^, we implemented sterile collection protocols and included matched environmental negative controls (air and saline swabs) to computationally filter contaminants using SourceTracker (0.9.1) and Decontam algorithms.

High-resolution transcriptomic profiling reveals different orbital fibroblast subpopulations and their gene expression programs associated with differentiation into adipocytes^13^. Concurrently, the data support the characterization of the local immune repertoire, with a focus on uncovering T-cell exhaustion signatures and defining macrophage polarization states within the orbital tissue^14,15^. Integrative analyses further provide a means to interrogate the existence of a different orbital microbiome and examine its statistical associations with specific host transcriptional programs. Our resources are essential for bridging the historical divide between cellular immunology and microbiology, thereby advancing the understanding of ocular autoimmunity.

It is important to note that the cellular and microbial profiles reported here derive from a Chinese patient cohort, and their interpretation should consider known ethnic and sociodemographic differences in TED presentation. A large multicenter study of 4,157 Chinese TED patients reported a mean age of 46.0 ± 16.4 years, a female-to-male ratio of approximately 1.8:1, and a predominance of moderate-to-severe disease (82.5%)^16^. These demographic features contrast with those of predominantly Western cohorts, which typically exhibit a higher female-to-male ratio (~6:1) and a greater proportion of mild disease. Furthermore, Chinese patients possess anatomically shallower orbits, resulting in lower mean Hertel exophthalmometry readings compared with Caucasian populations. These differences highlight the value of this Chinese dataset as a complement to existing Western cohorts and underscore the importance of population-specific reference ranges in TED research. Cross-ethnic comparative analyses leveraging both this and other publicly available datasets may yield insights into the genetic, environmental, and sociocultural determinants of TED pathogenesis and progression.

## Materials and Methods

### Sample collection and ethics

Orbital adipose tissue samples were collected from patients with TED and healthy controls at Beijing Tongren Hospital. The study was approved by the Ethics Committee of Beijing Tongren Hospital, Capital Medical University (Approval No. TRECKY2018-056-GZ (2022)-07) and was conducted in accordance with the principles of the Declaration of Helsinki, and written informed consent was obtained from all participants. The consent form covered voluntary participation, the scope of biological sample collection (orbital adipose tissue, conjunctival sac swabs, deep nasal swabs, and stool samples), the intended use for multi-omics research on thyroid eye disease, data storage for 10 years, and data sharing. Participants were informed that: (i) de-identified 16S rRNA sequencing data (bacterial sequences) would be deposited in a public repository (NCBI SRA); (ii) de-identified human single-cell RNA sequencing data (raw reads) would be deposited in a controlled-access repository (GSA-Human at CNCB); (iii) processed expression matrices would be deposited in an open-access repository (Zenodo); and (iv) no individually identifiable information would be shared. All participants provided consent for data sharing under these terms. Participants were enrolled at Beijing Tongren Hospital from January 2021 to December 2024. Patients with TED (diagnosed according to the Bartley diagnostic criteria) undergoing orbital decompression surgery were recruited as the case group. Control orbital and subcutaneous adipose tissue samples were collected from patients without thyroid disorders undergoing ocular plastic surgeries (for ptosis, single eyelids, or baggy eyelids). Enrollment was conducted by attending clinicians during routine preoperative consultations. Exclusion criteria included previous ocular surgery, concurrent metabolic diseases, and pregnancy or lactation. No participants from vulnerable populations were recruited, and no coercion was involved in the enrollment process. To minimize contamination for microbiome analysis, all surgeries were performed under aseptic conditions.

**Fig. 1:**
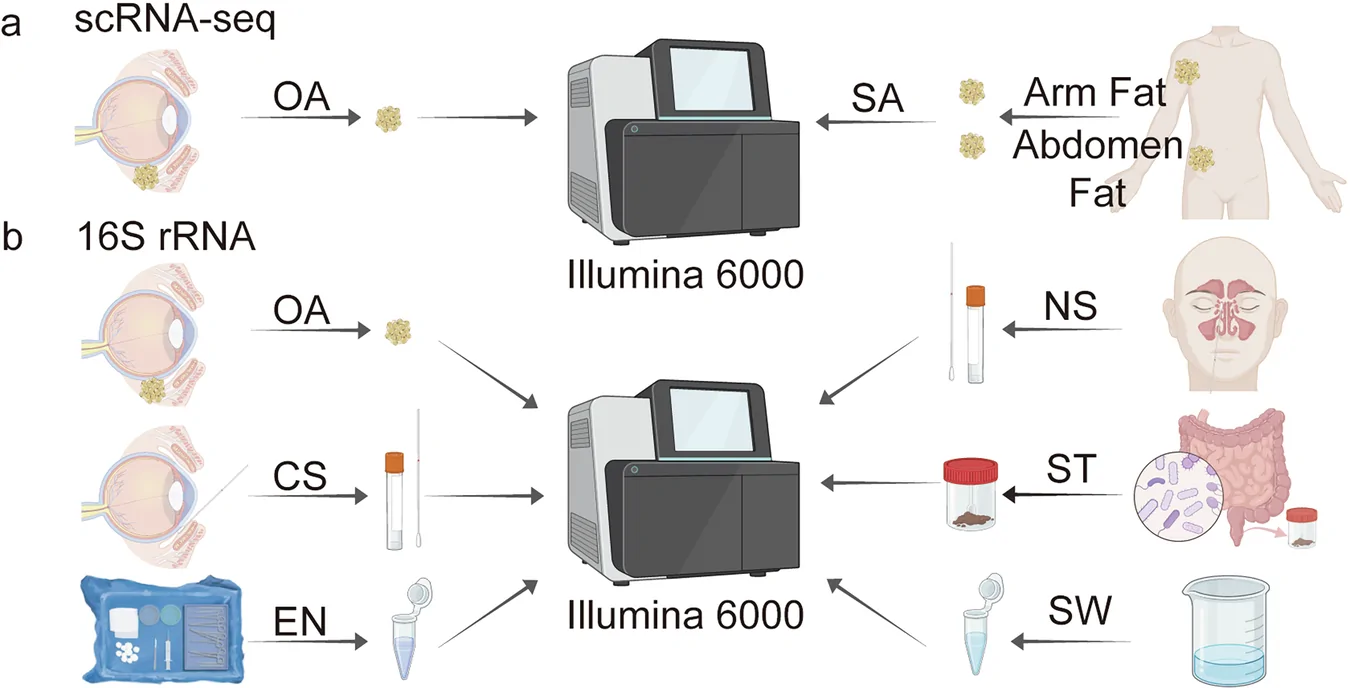
Sample collection and multi-omics profiling workflow. (**a**) Orbital adipose (OA) tissue samples were obtained from patients with thyroid eye disease (TED) undergoing orbital decompression surgery and from normal controls undergoing cosmetic eyelid surgery. In addition, subcutaneous adipose (SA) tissue, including abdominal and upper-arm fat, was collected. All tissue samples were enzymatically dissociated into single-cell suspensions, followed by library preparation and high-throughput single-cell RNA sequencing (scRNA-seq) using the 10x Genomics platform. (**b**) Orbital adipose tissue, conjunctival sac swabs (CS), deep nasal swabs (NS) and stool samples (ST) were collected from TED patients and normal controls. Negative controls were processed including environment samples (EN) and sterile water (SW). Genomic DNA was extracted from all samples, and libraries were prepared for 16S ribosomal RNA (16S rRNA) gene sequencing to characterize the microbial composition of the orbital microenvironment and related anatomical sites.

OA and additional subcutaneous adipose (SA) samples, including abdominal and upper-arm fat, were collected and enzymatically dissociated into single-cell suspensions for downstream scRNA-seq. For microbiome analysis, multiple sample types were collected from TED patients and controls, including OA, conjunctival sac swabs (CS), deep nasal swabs (NS), and stool samples (ST) (Fig. 1).

**Fig. 2:**
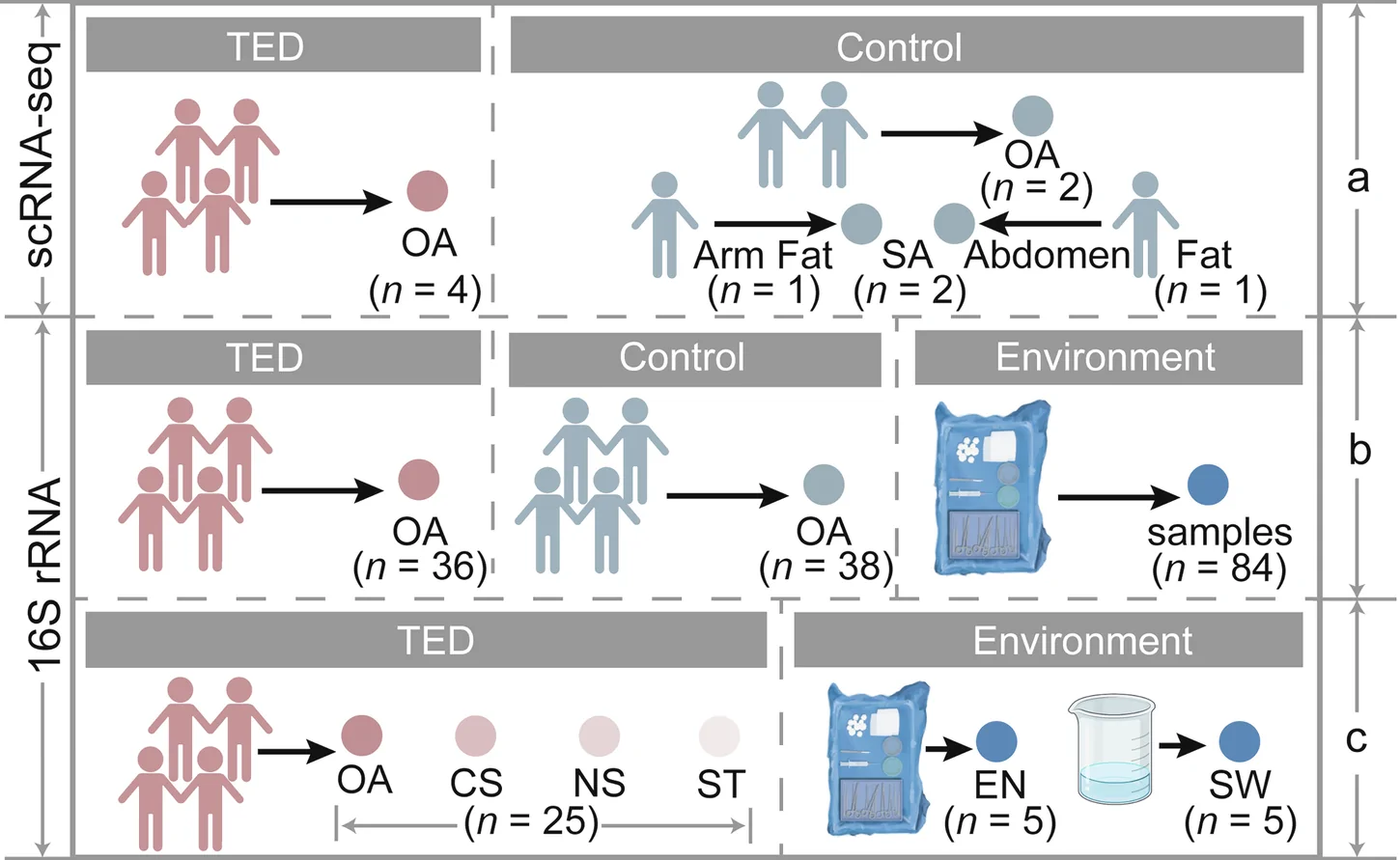
Grouping information. (**a**) Human orbital adipose tissue single-cell data from patients with thyroid eye disease (TED, *n* = 4) and controls (*n* = 4), collected via surgical procedures and stored at −80 °C. (**b**) Microbiome data from TED orbital adipose tissue (*n* = 36), environmental samples (*n* = 84), and healthy controls (*n* = 38), available in National Center for Biotechnology Information (NCBI) Sequence Archive SRP341968. (**c**) Data from 25 TED patients, with samples collected from orbital adipose tissue, conjunctival sac swabs, deep nasal swabs, and feces, plus 10 negative control samples.

Fresh OA samples were allocated for either scRNA-seq or 16S rRNA gene sequencing. For scRNA-seq, orbital adipose tissue samples from TED patients (*n* = 4) and controls (*n* = 4) were processed. Clinical metadata were extracted from electronic medical records, including age, sex, smoking status, thyroid function status at surgery, disease duration, prior thyroid and TED-specific treatments, and CAS (Table 1). Controls were confirmed to be euthyroid and free of thyroid disease. For microbiome profiling, orbital adipose tissue samples from TED patients (*n* = 36), along with environmental samples (*n* = 84) and healthy controls (*n* = 38), were included. A subset of these microbiome data has been previously reported in our earlier study^17^, in which we characterized the presence and composition of microbiota in orbital adipose tissue and implemented decontamination strategies using environmental controls. The integration of these previously published sequences with our newly generated datasets provides a resource for elucidating TED pathogenesis, with all reused data annotated under the GSA for human BioProject accession PRJCA067648. In addition, a separate cohort comprising 25 TED patients and 10 negative controls was used for multi-site microbiome sampling (OA, CS, NS, and stool) (Fig. 2 and Table 2).

**Table 1:** Clinical characteristics of the TED cohort and scRNA-seq subgroup. (a) Summary of the scRNA-seq TED subgroup (*n* = 4). (b) Summary of controls (*n* = 4). (c) Full TED cohort (*n* = 36) for context.

ID	Gender	Age	Clinical Activity Score (CAS)	Smoking History	Exophthalmos	Thyroid Status	Systemic Steroid	Orbital Radiation History	Thyroidec-tomy History
P1	Female	53	2/7	Secondhand smoke exposure	22 mm	Hyperthyroidism	Stopped 12 months prior	Never	Never
P2	Male	51	7/7	Active smoking	25 mm	Hyperthyroidism	Stopped 3 months prior	Never	Never
P3	Female	39	4/7	Secondhand smoke exposure	21 mm	Hyperthyroidism and Hashimoto’s thyroiditis	Stopped 6 months prior	Never	Never
P4	Female	42	2/7	Secondhand smoke exposure	23 mm	Hyperthyroidism	Stopped 6 months prior	Never	Never

Characteristic	Value
Age, mean (range)	42.5 years (22–74)
Sex, M:F	11:25 (1:2.3)
Smoking status, never n (%)	23 (64%)
Smoking exposure n (%)	13 (36%), Active smoking, 7 (19%), Secondhand smoke exposure, 6 (17%)
Hyperthyroidism, n (%)	30 (83%)
Prior antithyroid medication, n (%)	18 (50%)
Prior radioiodine therapy, n (%)	2 (5.6%)
Prior thyroidectomy, n (%)	1 (2.8%)
CAS ≥ 3 (active), n (%)	11 (31%)
Exophthalmos	21.8 mm (16–28 mm)

All four control participants were euthyroid individuals without thyroid disorders or TED, undergoing ocular plastic surgery or reconstructive surgery for non-inflammatory indications.

**Table 2:** Summary of study cohorts, sequencing modalities, and downstream analyses.

Cohort	Samples included	Sample size	Sequencing modality	Main analyses
Cohort 1 (scRNA-seq cohort)	Orbital adipose tissue from TED patients and healthy controls	TED (*n* = 4)	10x Genomics scRNA-seq	Cell clustering, cell-type annotation, differential expression analysis
		Healthy control (*n* = 4) (*n* = 4: orbital fat, *n* = 2; subcutaneous fat, *n* = 2)		
Cohort 2 (orbital adipose microbiome cohort)	TED orbital adipose tissue, environmental samples, and healthy controls	TED orbital adipose (*n* = 36)	16S rRNA sequencing (Illumina NovaSeq)	Microbiome profiling, contamination assessment, SourceTracker and Decontam analyses
		Environmental samples (*n* = 84)		
		Healthy controls (*n* = 38)		
Cohort 3 (multi-site microbiome cohort)	Orbital adipose tissue, conjunctival sac swabs, deep nasal swabs, and stool samples from TED patients	TED patients (*n* = 25; 100 samples in total: orbital adipose tissue, conjunctival sac swabs, deep nasal swabs, and stool samples)	16S rRNA sequencing (Illumina NovaSeq)	Cross-site microbiome comparison and community structure analysis
		10 negative controls (*n* = 10: sterile water, *n* = 5; empty collection tube controls, *n* = 5)		

To minimize potential contamination in microbiome analyses, all sampling and processing procedures were conducted under strict aseptic conditions. In parallel, multiple types of environmental and procedural controls were collected, including operating room air samples, surgical instruments, sampling swabs, collection tubes, and sterile buffer solutions, to monitor and account for potential contamination introduced during sampling and processing. These environmental controls were further used in downstream analyses to identify and remove potential contaminant signals.

We note that the clinical landscape for TED has shifted substantially since sample collection. The approval of the IGF-1 receptor antibody SYCUME® (teprotumumab N01) by China’s NMPA in March 2025 has led to a marked reduction in orbital decompression surgeries at our institution^18^. The specimens described in this study were collected prior to this transition and represent a pre-biologic-treatment surgical cohort.

### Single-cell RNA sequencing

Fresh tissue was enzymatically digested to generate single-cell suspensions. Cell viability and concentration were assessed prior to loading. Libraries were constructed using the Chromium Single Cell 3′ Library and Gel Bead Kit (10x Genomics)^19^, targeting a recovery of 9,000–10,000 cells per sample. Sequencing reads were demultiplexed to generate FASTQ files which were then aligned to the GRCh38 human reference genome using Cell Ranger (v10.0.0) to generate feature-barcode matrices.

### Sample collection and DNA extraction

Biological samples, including orbital adipose tissue, conjunctival sac swabs, deep nasal swabs, stool samples, and negative controls, were collected under sterile conditions and immediately transferred to sterile containers. All samples were stored at −80 °C until DNA extraction. To minimize potential contamination, low-biomass samples and high-biomass samples were processed separately, and DNA extraction and PCR were performed using independent plates. Sample positions were randomized, and sample identifiers were scrambled during sequencing.

### 16S rRNA gene sequencing

Total genomic DNA was extracted from frozen tissue samples, swab samples, stool samples, and negative controls using a sterile DNA extraction kit according to the manufacturer’s instructions. The V3–V4 hypervariable regions of the bacterial 16S rRNA gene were amplified using the universal primers 338 F (5′-ACTCCTACGGGAGGCAGCA-3′) and 806 R (5′-GGACTACHVGGGTWTCTAAT-3′)^20^. Amplicons were purified and pooled at equimolar concentrations to construct sequencing libraries. Library concentration and fragment size distribution were assessed using Qubit 4.0 fluorometry and Qsep400 capillary electrophoresis prior to sequencing. Multi-omics-end sequencing (2 × 250 bp) was performed on the Illumina NovaSeq 6000 platform. Raw sequencing data were generated in multi-omics-end FASTQ format and subjected to initial quality control by the sequencing provider.

### Data processing

For scRNA-seq, downstream analysis was performed using the Seurat (v5.3.1) R package^21^. Quality control involved filtering cells based on mitochondrial gene content (>15% excluded) and gene counts (<200 or >8,000 detected genes excluded). Putative doublets were identified and removed using scDblFinder (v2.0.4). Dimensionality reduction (PCA, UMAP) and clustering were performed to identify cell types. Cell-type annotation was performed using canonical marker genes. To provide comprehensive marker gene information, differential expression analysis was performed using Seurat’s FindAllMarkers function with the following parameters: Wilcoxon rank-sum test, only.pos = TRUE, min.pct = 0.25, logfc.threshold = 0.25. The top 20 marker genes ranked by average log2 fold-change were selected as representative markers for each cluster. Annotation confidence was assessed manually based on the specificity and established nature of marker gene expression.

For microbiome data, raw reads were processed using the QIIME 2 (version 2025.10) pipeline^22^. Denoising and amplicon sequence variant (ASV) calling were performed using DADA2^23^. Taxonomy was assigned using the SILVA database^24^. Potential contaminants were identified and removed using the Decontam^25^ R package and SourceTracker^26^, utilizing the environmental negative controls as the source of background noise.

## Data Records

All raw sequencing data and processed datasets generated in the study have been deposited in publicly accessible repositories. Below we provide the accession numbers for each read set and their correspondence to individual libraries.

Single-cell RNA sequencing data. Raw scRNA-seq FASTQ files for all eight samples (four TED: Orbit_P1–P4; four controls: Orbit_N1–N2, Abdomen_N, Armpit_N) are available under controlled access at the Genome Sequence Archive for Human (GSA-Human) at the China National Center for Bioinformation (CNCB), under accession HRA019310^27^. Individual sample-level accessions within this project are listed in Table 3.

**Table 3:** Overview of single-cell RNA sequencing data deposition and sample-level accessions.

Sample ID	Group	Tissue	Repository	GSA-Human Accession: [HRA019310]^27^
Abdomen_N	Control	Abdominal subcutaneous fat	GSA-Human (CNCB)	[HRI1968418]
Armpit_N	Control	Upper-arm subcutaneous fat	GSA-Human (CNCB)	[HRI1968419]
Orbit_N1	Control	Orbital adipose tissue	GSA-Human (CNCB)	[HRI1968416]
Orbit_N2	Control	Orbital adipose tissue	GSA-Human (CNCB)	[HRI1968417]
Orbit_P1	TED	Orbital adipose tissue	GSA-Human (CNCB)	[HRI1968412]
Orbit_P2	TED	Orbital adipose tissue	GSA-Human (CNCB)	[HRI1968413]
Orbit_P3	TED	Orbital adipose tissue	GSA-Human (CNCB)	[HRI1968414]
Orbit_P4	TED	Orbital adipose tissue	GSA-Human (CNCB)	[HRI1968415]

Processed scRNA-seq gene–cell count matrices in Market Exchange Format (.mtx), together with barcode and feature annotation files, have been deposited in Zenodo under https://doi.org/10.5281/zenodo.18983916^28^.

16S rRNA gene sequencing data. Raw 16S rRNA amplicon sequencing data (V3–V4 region, Illumina NovaSeq 6000, 2 × 250 bp) are publicly available at the NCBI Sequence Read Archive (SRA) under SRA Study Accessions SRP682191^29^ and SRP682192^30^ (Table 4).

**Table 4:** Overview of 16S rRNA gene sequencing data deposition and sample-level accessions.

Dataset Description	SRA Study accession	SRX Study Accession	No. of Samples
Orbital adipose tissue microbiome (TED *n* = 36, healthy controls *n* = 38, environmental samples *n* = 84)	[SRP341968]^31^	[SRX12677182] — [SRX12677339]	158
Multi-site cohort — 25 OA samples	[SRP682191]^29^	[SRX32420213] — [SRX32420237]	25
Multi-site cohort —25 conjunctival swab, 25 nasal swab, 25 stool, 5 sterile water, 5 empty collection tube samples	[SRP682192]^30^	[SRX32420238] — [SRX32420322]	85

A subset of the orbital adipose tissue 16S rRNA data (SRP341968^31^) was previously reported in our earlier study^17^. Taxon × sample tables and related supplementary data are available as supporting information in that publication.

Processed microbiome feature tables, including the decontaminated amplicon sequence variant (ASV) table and corresponding taxonomic annotations (from phylum to genus level), have been deposited in Zenodo under https://doi.org/10.5281/zenodo.18385494^32^ and https://doi.org/10.5281/zenodo.18983916.

All datasets are accessible for reproducibility and further analyses. A consolidated data index is provided in Table 5.

**Table 5:** Inventory of deposited datasets.

Dataset	Repository	Accession / DOI	Data Type	Processing Level	Recommended Use
scRNA-seq raw reads (TED + control)	GSA for Human	HRA019310^27^	FASTQ	Raw	Re-alignment, alternative mapping pipelines
scRNA-seq gene–cell matrices	Zenodo	https://doi.org/10.5281/zenodo.18983916^28^	matrix / barcodes / features	Processed	Cell clustering, differential expression, trajectory analysis
16S rRNA raw reads	NCBI SRA	SRP341968^31^	FASTQ	Raw	Re-processing with alternative denoising parameters
16S rRNA raw reads (multi-site samples from TED)	NCBI SRA	SRP682191^29^ (orbital adipose) SRP682192^30^ (other samples)	FASTQ	Raw	Re-processing, cross-site comparison
Decontaminated ASV table + taxonomy	Zenodo	https://doi.org/10.5281/zenodo.18983916^28^ https://doi.org/10.5281/zenodo.18385494^32^	Feature table (.qza/.tsv/.xls)	Processed	Alpha/beta diversity, differential abundance, correlation analyses

## Data Overview

Using the 10x Genomics Chromium platform, we generated single-cell RNA sequencing data from human orbital adipose tissue samples collected from patients with TED and healthy controls. After quality filtering and aggregation, a total of 61,302 high-quality cells were retained for downstream analyses. The single-cell transcriptomes exhibited robust sequencing depth, with consistent numbers of detected genes and unique molecular identifiers per cell across samples, supporting reliable characterization of major stromal and immune cell populations.

**Fig. 3:**
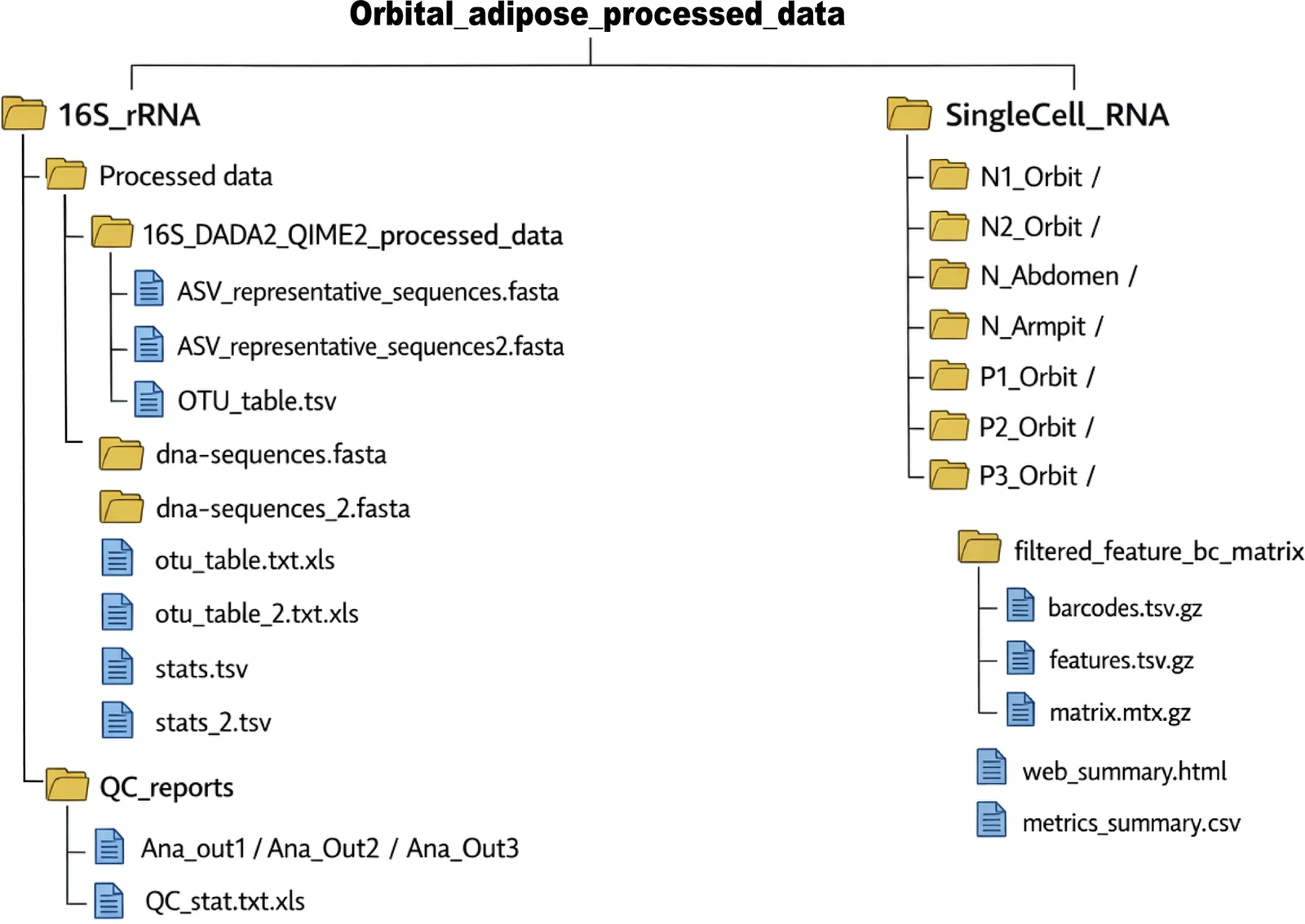
Hierarchical structure of the data deposited in https://doi.org/10.5281/zenodo.18983916 labeled Orbital_Adipose_processed_data.zip. The dataset contains 16S ribosomal RNA (16S rRNA) sequencing data and single-cell RNA sequencing (scRNA-seq) data, organized into processed data, quality control reports, and sample-specific folders.

In parallel, 16S rRNA gene sequencing targeting the V3–V4 hypervariable regions was performed on matched orbital adipose tissue samples and environmental negative controls. Sequencing data showed high retention of effective reads following quality filtering and denoising, and a substantial proportion of reads could be assigned at the phylum level after contamination-aware processing. Together, these datasets constitute a high-quality multi-omics resource for investigating the cellular landscape and potential microbial signatures of the orbital microenvironment in thyroid eye disease (Fig. 3).

## Technical Validation

### scRNA-seq quality control

Single-cell RNA sequencing (scRNA-seq) libraries were generated using the 10x Genomics Chromium platform with the Single Cell 3′ v3 chemistry. Cell quality and concentration were assessed prior to library construction, and libraries were sequenced on an Illumina NovaSeq 6000 platform with paired-end 150 bp reads, with sufficient sequencing depth for downstream analyses. Raw sequencing data were processed using Cell Ranger (v10.0.0) with the GRCh38 reference transcriptome, and samples were aggregated to normalize sequencing depth across samples.

**Fig. 4:**
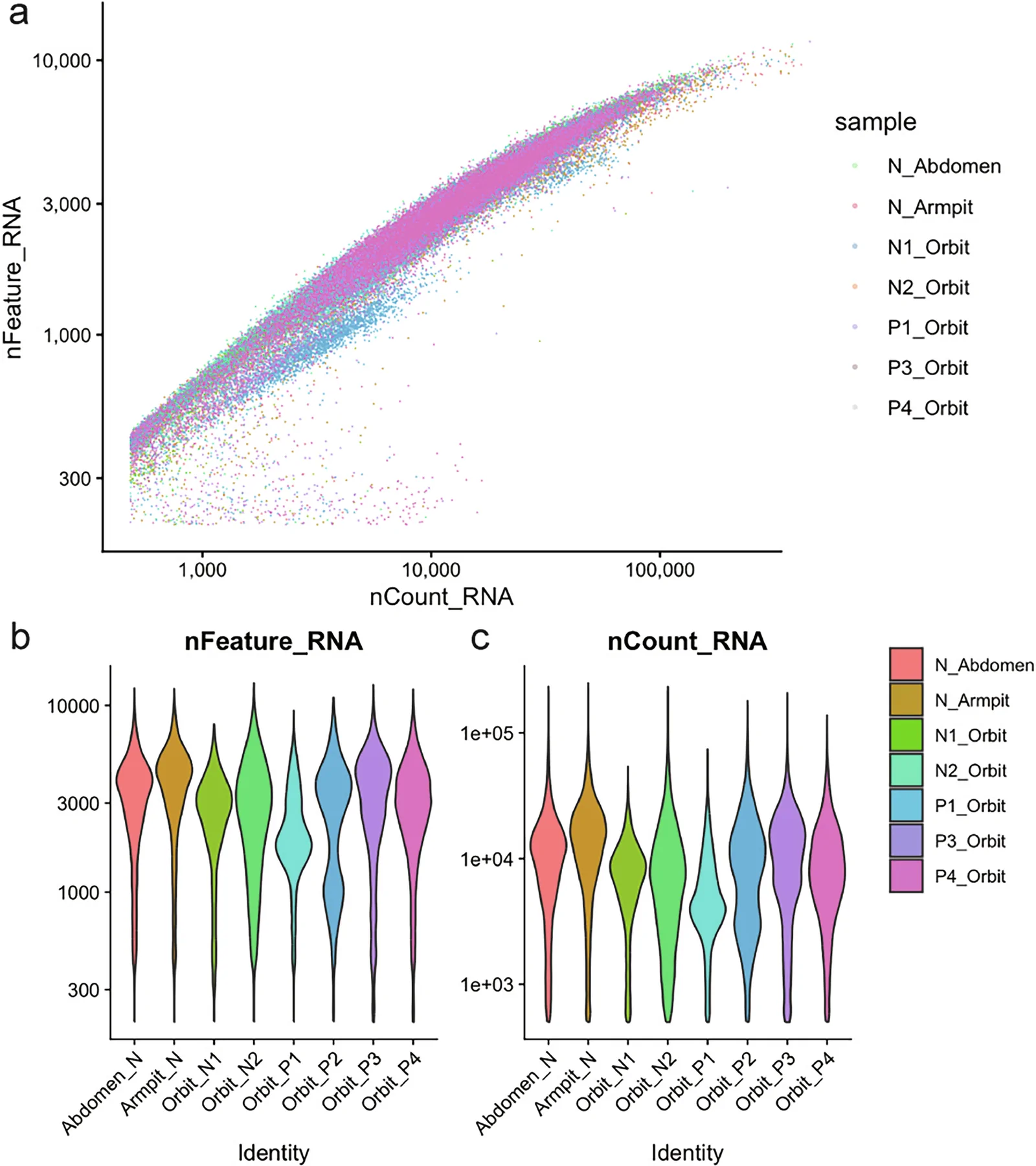
Single-cell RNA-sequencing (scRNA-seq) data quality control. (**a**) Scatter plot of number of detected genes per cell (nGene) versus unique molecular identifier(s) nUMI for all cells, showing the correlation between gene detection and sequencing depth. (**b**) Violin plots of nCount_RNA (total UMI counts per cell) across samples. (**c**) Violin plots of nFeature_RNA (number of detected genes per cell) across samples.

According to the aggregated Cell Ranger report, a total of 72,728 cells were captured across all samples, with an average of 86.8% of sequencing reads assigned to cell-associated barcodes, indicating efficient cell capture and low ambient RNA background. The median number of detected genes per cell was approximately 3,700, with values ranging from 2,644 to 6,906, reflecting expected variability across different cell types. The median UMI counts per cell were approximately 13,700, ranging from 6,146 to 28,966, indicating sufficient sequencing depth and transcriptomic complexity. (Table 6) Quality metrics of single cells, including the relationship between detected genes and UMI counts, as well as the distributions of total UMI counts (nCount_RNA) and detected genes (nFeature_RNA) per cell, demonstrated overall data quality and consistency across samples (Fig. 4). Downstream quality control was performed using the Seurat (v5.3.1) R package, during which cells with fewer than 200 detected genes, cells with more than 8,000 detected genes, or cells with mitochondrial gene content exceeding 15% were removed. Potential doublets were identified and removed using scDblFinder (v2.0.4) (Table 7). After stringent quality filtering, a total of 61,302 high-quality cells were retained for downstream analyses. (Table 8) (Table 9).

**Table 6:** Quality control of scRNA-seq data based on cells.

Sample	Estimated number of cells	Fraction reads in cells (%)	Mean reads per cell	Median genes per cell	Total genes detected	Median UMI counts per cell
Abdomen_N	9193	88.90	67589	3232	30543	9410
Armpit_N	8895	85.70	70115	3922	31472	13258
Orbit_N1	5996	85.60	23991	6906	26520	27987
Orbit_N2	12218	89.20	47473	2644	30402	6146
Orbit_P1	7542	86.20	17022	4224	18906	28966
Orbit_P2	8903	87.20	66598	2767	30967	7626
Orbit_P3	8017	81.30	70997	3192	31041	9125
Orbit_P4	11964	89.80	50555	2850	31776	7378

**Table 7:** Summary of cell numbers, quality-control filtering, and doublet removal across all scRNA-seq samples.

Sample	Cells input	Cells after QC	QC removal rate (%)	Predicted doublets	Doublet rate (%)	Singlets retained
N1	5996	5404	9.87	421	7.79	4983
N2	12218	11640	4.73	1650	14.18	9990
N_abdomen	9193	8497	7.57	722	8.50	7775
N_arm	8895	8300	6.69	745	8.98	7555
P1	7542	7264	3.69	637	8.77	6627
P2	8903	8527	4.22	638	7.48	7889
P3	8017	7527	6.11	662	8.79	6865
P4	11964	10863	9.20	1245	11.46	9618
**Total**	**72728**	**68022**	**6.47**	**6720**	**9.88**	**61302**

**Note:** Cells were filtered using the following quality-control criteria: nFeature_RNA > 200, nFeature_RNA < 8,000, and mitochondrial transcript content (percent.mt) < 15%. Putative doublets were identified and removed using scDblFinder (v2.0.4) with default parameters. Doublet rate was calculated as the percentage of predicted doublets among cells retained after quality-control filtering.

**Table 8:** Quality control of raw scRNA-seq data on sequencing.

Sample	Number of reads	Number of short reads skipped	Valid barcodes (%)	Valid UMIs (%)	Sequencing saturation (%)	Q30 bases in barcode (%)	Q30 bases in RNA read (%)	Q30 bases in UMI (%)
Abdomen_N	621,347,258	0	97.50	100.00	74.40	97.30	93.80	97.00
Armpit_N	623,671,689	0	96.90	99.90	58.40	97.10	92.50	96.90
Orbit_N1	143,850,553	0	98.20	99.90	53.80	96.90	93.50	96.70
Orbit_N2	580,021,576	0	97.70	100.00	71.30	96.80	92.80	96.20
Orbit_P1	128,381,666	0	98.30	99.90	48.90	96.80	93.20	96.70
Orbit_P2	592,919,612	0	96.80	99.90	69.90	95.90	87.90	94.00
Orbit_P3	569,179,763	0	97.00	99.90	67.40	95.80	87.90	94.00
Orbit_P4	604,837,387	0	97.10	100.00	65.50	97.20	93.40	96.90

**Table 9:** Quality control of raw scRNA-seq data on mapping.

Sample	Reads mapped to genome (%)	Mapped confidently to genome (%)	Confidently to intergenic (%)	Confidently to intronic (%)	Confidently to exonic (%)	Confidently to transcriptome (%)	Reads mapped antisense to gene (%)
Abdomen_N	96.50	93.00	5.60	23.90	63.50	77.30	9.30
Armpit_N	92.10	85.10	7.20	24.10	53.80	68.60	8.50
Orbit_N1	96.70	92.80	6.20	17.10	69.40	81.10	4.60
Orbit_N2	96.40	92.60	5.40	24.60	62.60	76.40	9.80
Orbit_P1	92.10	85.10	7.20	24.10	53.80	68.60	8.50
Orbit_P2	90.80	85.20	7.10	27.70	50.40	67.90	9.60
Orbit_P3	91.70	83.10	6.70	22.00	54.40	67.30	8.40
Orbit_P4	96.60	88.80	8.90	28.70	51.10	66.30	12.90

### Cell clustering and annotation

**Fig. 5:**
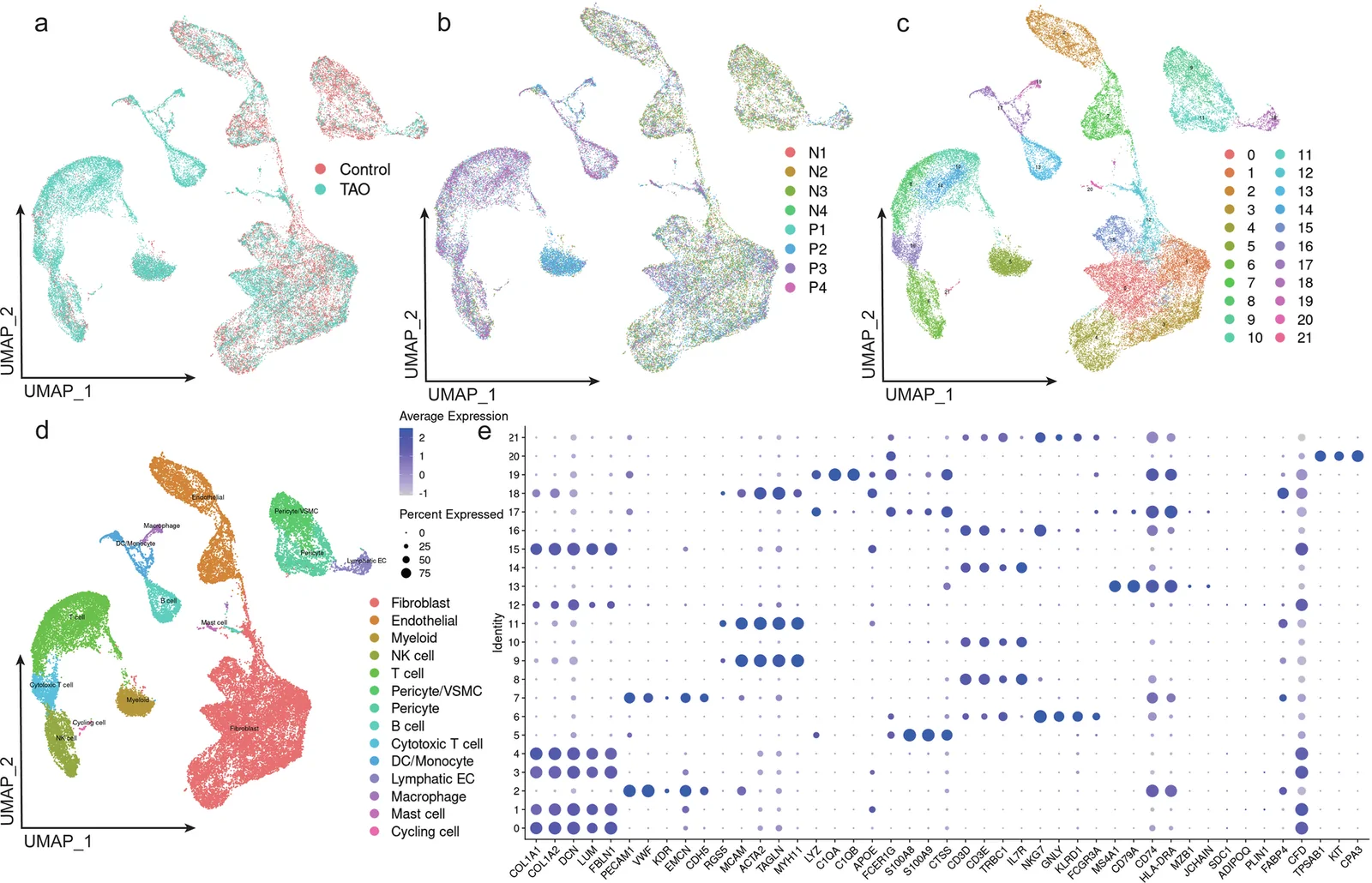
Cell clustering and gene expression patterns of single-cell RNA sequencing (scRNA-seq) samples. **(a)** Uniform Manifold Approximation and Projection (UMAP) visualization of cells colored by sample group: orbit-N (orbital adipose tissue from healthy controls) and orbit-P (orbital adipose tissue from patients with thyroid eye disease, TED). **(b)** UMAP visualization of all cells colored by sample origin (N1–N4 and P1–P4, N3 = Abdomen_N, N4 = Armpit_N), demonstrating sample integration and the distribution of cells across donors. **(c)** UMAP visualization of unsupervised clustering showing 22 transcriptionally distinct clusters (clusters 0–21). **(d)** UMAP annotated with major cell types, including fibroblast, endothelial, myeloid, T cell, NK cell, pericyte/vascular smooth muscle cell (VSMC), B cell, cytotoxic T cell, dendritic/monocyte, lymphatic endothelial cell, macrophage, mast cell, and cycling cell populations. Cell-type assignments were inferred based on the expression of canonical marker genes (Table 10) and are supported by the multi-gene expression patterns shown in panel e. **(e)** Dot plot of canonical marker gene expression across clusters. Dot size represents the percentage of cells expressing each gene; color intensity represents the average log1p-transformed (log[1 + x]) normalized expression level, respectively. The coordinated expression of multiple markers per cell type confirms the UMAP-based annotations in panel d. Cell-type annotations are based on canonical marker expression and should be considered as high-confidence assignments for broad categories (fibroblast, T cell, myeloid, endothelial, NK, macrophage, epithelial). Finer sub-annotations, particularly within fibroblast and T-cell compartments, are intended as reference points that would benefit from independent validation, as noted in the Usage Notes.

To further evaluate the biological structure and reliability of the dataset, dimensionality reduction and clustering analyses were performed. Cells were well distributed in the UMAP space and formed distinct clusters, indicating pronounced cellular heterogeneity within orbital adipose tissue. Based on canonical marker gene expression, major cell populations were identified and annotated. Stromal fibroblasts were characterized by high expression of extracellular matrix-associated genes such as COL1A2, DCN, and FBLN1, forming several prominent clusters. Vascular-associated cells, including endothelial cells, were identified by the expression of PECAM1, VWF, and ECSCR. Immune cell populations were also clearly resolved, with T cells expressing markers such as TRBC2 and SKAP1, and myeloid cells exhibiting expression of genes including CD52 and C3. Additional cell populations, including epithelial or gland-associated cells, were supported by marker genes such as AQP1 and SERPINF1. These cell types exhibited different and cluster-specific expression patterns, supporting the robustness of the broad cell-type annotations while preserving the continuous transcriptomic states inherent to tissue remodeling in TED. The distribution of clusters was consistent across samples, and the observed transcriptional signatures aligned with known biological identities of orbital tissue-resident and infiltrating cells. Together, these results demonstrate that the dataset captures the complex cellular composition of orbital adipose tissue and provides a robust foundation for downstream analysis of stromal, vascular, and immune compartments (Fig. 5).

To facilitate detailed evaluation of cell-type annotation quality, we performed differential expression analysis using Seurat’s FindAllMarkers (Wilcoxon rank-sum test; only.pos = TRUE, min.pct = 0.25, logfc.threshold = 0.25) and identified the top 20 representative markers for each of the 22 transcriptionally distinct clusters. The complete annotation, including assigned cell identities, annotation confidence, and marker genes, is provided in Table 10. Additionally, a DotPlot of 45 canonical cell-type marker genes across all major clusters is provided in Fig. 5e to complement the broad annotations shown in Fig. 5d.

**Table 10:** Cell-cluster annotation summary.

Cluster	Assigned cell identity	Representative marker genes	Confidence
0	Fibroblast	SFRP2, RSPO2, PI16, PTN, THSD4	High
1	Fibroblast	SMOC1, BMPER, ADAM12, SEMA3A, PLA2G2A	High
2	Endothelial cell	ACKR1, SELE, SELP, PLVAP, AQP1	High
3	Fibroblast	DDR2, GFPT2, PLA2G2A, HAS2, ADAM12	High
4	Fibroblast	PRG4, PTHLH, DACT2, PAMR1, ERBB3	High
5	Neutrophil	FCGR3B, CYP4F3, AQP9, PROK2, TREM1	High
6	NK cell	GNLY, FGFBP2, GZMB, KLRF1, KLRC2	High
7	Arterial endothelial cell	SOX17, GJA5, SEMA3G, DLL4, IGFBP3	High
8	Activated T cell	ICOS, CD28, IL7R, DUSP4, FAAH2	High
9	Vascular smooth muscle cell	MYH11, MYOCD, SLC22A3, HS3ST2, TNC	High
10	Naïve/Central memory T cell	CCR7, LEF1, MAL, TESPA1, CAMK4	High
11	Pericyte	RGS5, ENTPD3, NDUFA4L2, CASQ2, COX4I2	High
12	PI16^+^ fibroblast	PI16, SFRP2, COL1A1, COL1A2, DCN	High
13	B cell	CD79A, PAX5, IGHD, FCRLA, TCL1A	High
14	T cell	IL7R, LEF1, TCF7, SLAMF1, CAMK4	High
15	Fibroblast (CRABP1^+^ subtype)	CRABP1, HHIP, GDF10, MYOC, IGF1	Moderate
16	CD8^+^ T cell	CD8A, CD8B, GZMK, CCL5, CRTAM	High
17	Dendritic cell/Monocyte	CD1C, CD86, CLEC10A, FCN1, LYZ	High
18	Lymphatic endothelial cell	CCL19, CCL21, EGFL6, SSTR2, STEAP4	High
19	Macrophage	C1QA, C1QB, C1QC, FOLR2, CD163	High
20	Mast cell	TPSAB1, TPSB2, CMA1, CPA3, KIT	High
21	Cycling cell	MKI67, KIF18B, RRM2, NEK2, HJURP	High

### 16S rRNA microbiome quality control

In the multi-site microbiome cohort, a total of 110 samples were included for 16S rRNA gene sequencing. After quality filtering and chimera removal, the average number of raw reads per sample was 100,951, with a median of 115,331 and a range from 2,764 to 126,692. The sequencing data were of high quality, with a mean Q30 score of 95.50%, a median of 95.60%, and values ranging from 94.00% to 96.60% across all samples. Following denoising and chimera filtering using the DADA2 pipeline, an average of 86.30% of reads per sample were retained as high-quality non-chimeric sequences, with a median of 91.56% and a range from 45.45% to 95.08%. Samples from conjunctival sac swabs exhibited relatively lower sequencing depth compared to other sample types, which is consistent with the low microbial biomass characteristic of the ocular surface^33^. This variation in sequencing depth across anatomical sites reflects inherent biological differences in microbial load rather than technical bias. To minimize potential bias arising from differences in sequencing depth, all downstream analyses were performed on normalized data. Inclusion of all samples did not affect overall diversity patterns or the robustness of the results.

To ensure the reliability of microbiome data and minimize the impact of potential contamination, multiple types of negative controls were included throughout sample collection and processing, including sterile water controls and environmental samples such as air exposure, surgical instruments, and sampling materials. DNA extraction and library preparation were performed in parallel for biological samples and negative controls. Sterile water controls consistently exhibited markedly lower alpha diversity compared with biological samples, reflecting minimal background contamination. In contrast, environmental controls displayed relatively higher diversity, which is expected given their exposure to ambient microbial sources. Importantly, beta diversity analysis based on Bray–Curtis dissimilarity demonstrated that microbial community structures of environmental controls were clearly distinct from those of biological samples, indicating that environmental signals did not overlap with or dominate the microbial profiles observed in biological specimens. Together, these results support effective discrimination between background contamination and putative tissue-associated microbial DNA signals. The inclusion of multiple negative control types enabled systematic monitoring of potential contamination introduced during sample handling and sequencing, following established practices for low-biomass microbiome studies, thereby strengthening the reliability and interpretability of the microbiome dataset. Detailed per-sample sequencing statistics are provided in Table 11.

**Table 11:** Summary of 16S rRNA sequencing quality control metrics for all samples.

sample-id	input	filtered	percentage of input passed filter	denoised	non-chimeric	percentage of input non-chimeric
SW1	115085	109161	94.85	108034	106140	92.23
SW2	113203	107399	94.87	106305	104731	92.52
SW3	116181	110861	95.42	101136	93886	80.81
SW4	115637	109472	94.67	108441	107149	92.66
SW5	120269	113030	93.98	111706	109928	91.4
T–EN1	115530	110951	96.04	110315	109625	94.89
T–EN2	42151	40142	95.23	39860	39604	93.96
T–EN3	106604	102691	96.33	101955	101045	94.79
T–EN4	10685	10171	95.19	10058	10024	93.81
T–EN5	103555	99129	95.73	98533	97328	93.99
T001AD	111837	107956	96.53	105805	99796	89.23
T001CS	120878	116133	96.07	115303	114233	94.5
T001NS	113805	109821	96.5	107039	104182	91.54
T001ST	123144	117517	95.43	111625	92319	74.97
T002AD	115403	110550	95.79	108759	106423	92.22
T002CS	119239	113484	95.17	112903	111936	93.88
T002NS	115484	111051	96.16	108596	105887	91.69
T002ST	123245	118114	95.84	106903	72366	58.72
T003AD	111205	105054	94.47	103138	100649	90.51
T003CS	3452	3252	94.21	3061	3061	88.67
T003NS	122453	118587	96.84	116725	106020	86.58
T003ST	122989	117983	95.93	113806	99800	81.15
T004AD	108646	104129	95.84	101834	100767	92.75
T004CS	59832	56918	95.13	56343	56013	93.62
T004NS	116680	112041	96.02	109873	102493	87.84
T004ST	121885	117260	96.21	105784	55400	45.45
T005AD	108098	104172	96.37	101217	99639	92.17
T005CS	50129	47814	95.38	47313	46667	93.09
T005NS	115226	111533	96.79	108863	107533	93.32
T005ST	126025	121261	96.22	110772	77384	61.4
T006AD	96846	93762	96.82	91503	90986	93.95
T006CS	43927	41854	95.28	41376	41150	93.68
T006NS	114574	110769	96.68	108171	104666	91.35
T006ST	120855	116391	96.31	107656	70650	58.46
T007AD	105177	102105	97.08	99604	98401	93.56
T007CS	68205	65446	95.95	64820	64182	94.1
T007NS	115807	110999	95.85	108963	106138	91.65
T007ST	125996	120944	95.99	113995	86910	68.98
T008AD	115047	109097	94.83	107105	105192	91.43
T008CS	2764	2609	94.39	2435	2435	88.1
T008NS	122939	118004	95.99	116352	109981	89.46
T008ST	118023	113648	96.29	108469	90657	76.81
T009AD	103799	100171	96.5	97435	94687	91.22
T009CS	10289	9844	95.67	9588	9077	88.22
T009NS	114244	109262	95.64	107657	103666	90.74
T009ST	116000	111402	96.04	104827	74751	64.44
T010AD	108898	104415	95.88	101636	97667	89.69
T010CS	67074	64189	95.7	63596	63333	94.42
T010NS	119918	114196	95.23	111800	108554	90.52
T010ST	122036	117770	96.5	113798	99910	81.87
T011AD	104674	101506	96.97	98882	98250	93.86
T011CS	122165	117559	96.23	116698	115972	94.93
T011NS	123537	118886	96.24	117139	104602	84.67
T011ST	124158	119170	95.98	110335	85508	68.87
T012AD	109790	104745	95.4	102477	101509	92.46
T012CS	119696	113811	95.08	112966	112206	93.74
T012NS	122622	116829	95.28	115291	111226	90.71
T012ST	116173	111520	95.99	107870	87176	75.04
T013AD	114897	110126	95.85	107523	105693	91.99
T013CS	121371	116935	96.35	116325	115397	95.08
T013NS	124182	119255	96.03	118430	116320	93.67
T013ST	124312	120009	96.54	114392	76434	61.49
T014AD	112883	107433	95.17	104912	102783	91.05
T014CS	7554	7186	95.13	6952	6932	91.77
T014NS	113766	107273	94.29	105873	102161	89.8
T014ST	124969	119328	95.49	110824	63744	51.01
T015AD	110264	105995	96.13	103593	102589	93.04
T015CS	5206	5005	96.14	4838	4821	92.6
T015NS	125297	120041	95.81	118927	108656	86.72
T015ST	123961	119304	96.24	112358	73163	59.02
T016AD	111987	106674	95.26	104392	102510	91.54
T016CS	120321	114032	94.77	113301	112235	93.28
T016NS	118454	113733	96.01	112061	109239	92.22
T016ST	122902	117886	95.92	112682	84968	69.13
T017AD	94649	91394	96.56	89162	88083	93.06
T017CS	8410	8021	95.37	7792	7754	92.2
T017NS	123341	118385	95.98	117355	112108	90.89
T017ST	122651	118109	96.3	110335	80175	65.37
T018AD	58344	56927	97.57	54292	54029	92.6
T018CS	55096	52436	95.17	51908	51573	93.61
T018NS	120414	114539	95.12	112812	104419	86.72
T018ST	123313	118365	95.99	111742	81843	66.37
T019AD	113914	110447	96.96	107569	106489	93.48
T019CS	5861	5617	95.84	5424	5400	92.13
T019NS	122169	117783	96.41	117087	112486	92.07
T019ST	126041	121606	96.48	118513	101923	80.86
T020AD	109441	105735	96.61	103104	101588	92.82
T020CS	12552	11970	95.36	11728	11698	93.2
T020NS	119431	114038	95.48	112181	105322	88.19
T020ST	124343	119307	95.95	115502	97051	78.05
T021AD	117172	111915	95.51	109623	108617	92.7
T021CS	85435	81641	95.56	81104	80540	94.27
T021NS	117399	112842	96.12	109852	103040	87.77
T021ST	125210	119601	95.52	112965	91764	73.29
T022AD	112319	109245	97.26	105586	104742	93.25
T022CS	79651	76342	95.85	75721	75221	94.44
T022NS	122969	117801	95.8	113756	108066	87.88
T022ST	125468	119925	95.58	114076	88933	70.88
T023AD	110410	105748	95.78	102604	101111	91.58
T023CS	95671	91755	95.91	91136	89904	93.97
T023NS	124135	117894	94.97	117257	109962	88.58
T023ST	126692	121870	96.19	112952	87167	68.8
T024AD	57488	56089	97.57	53618	53366	92.83
T024CS	14717	14135	96.05	13923	13820	93.91
T024NS	115259	110657	96.01	109088	104546	90.71
T024ST	121435	116423	95.87	110897	83907	69.1
T025AD	111643	108013	96.75	105248	102572	91.87
T025CS	70588	68005	96.34	67319	66947	94.84
T025NS	99387	95668	96.26	93200	92392	92.96
T025ST	125515	120161	95.73	113248	78729	62.72

**Note:** AD, orbital adipose tissue; CS, conjunctival sac swab; NS, deep nasal swab; ST, stool; SW, sterile water negative control; T–EN, tube–environmental controls.

### Microbial community structure and validation

**Fig. 6:**
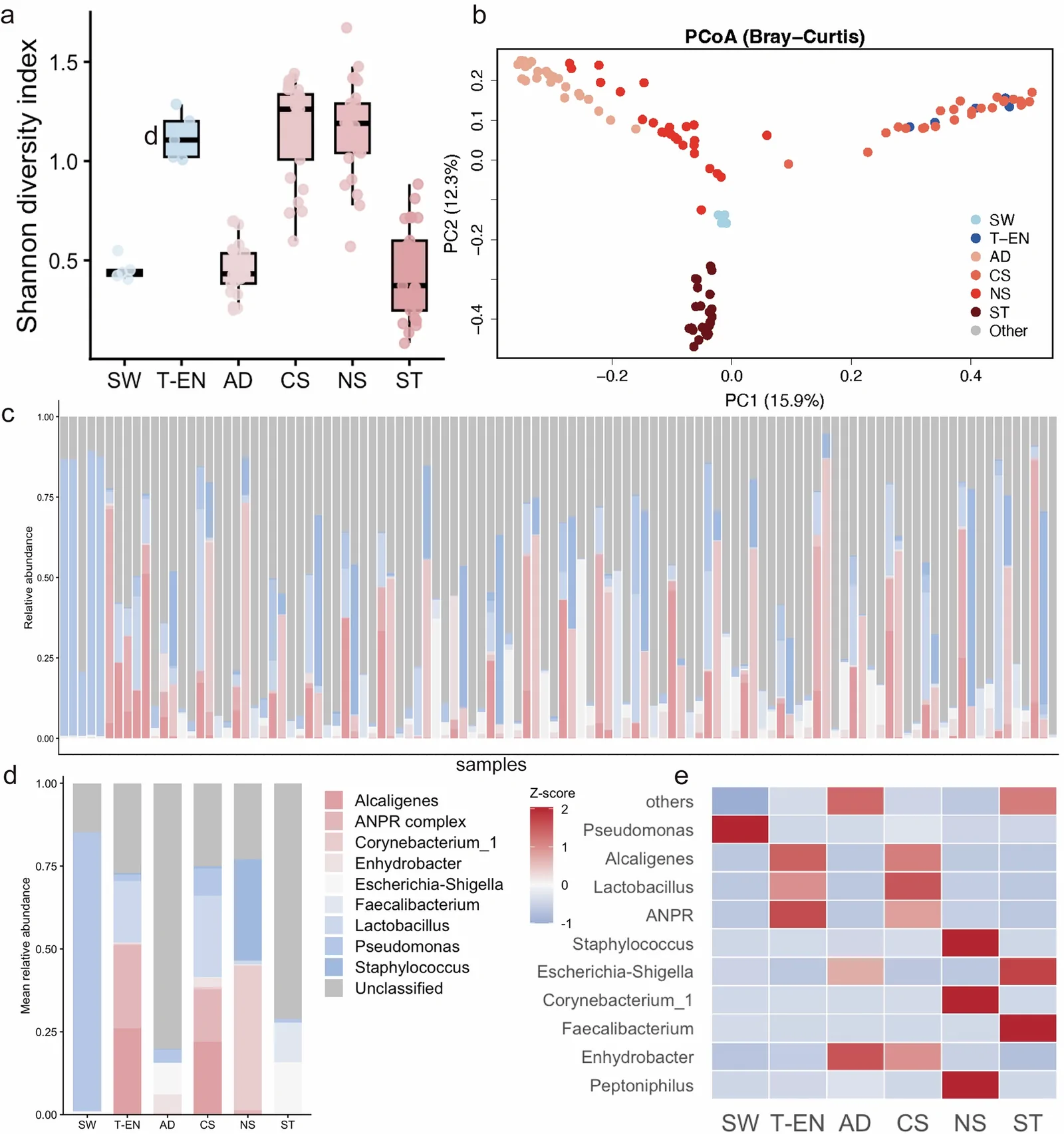
Microbial community structure and validation of the orbital adipose tissue 16S ribosomal RNA (16S rRNA) dataset. **(a)** Alpha diversity assessed using the Shannon diversity index. Sterile water controls exhibited markedly lower diversity compared with biological samples, supporting effective discrimination between background contamination and biological microbial signals. (**b**) Principal coordinate analysis (PCoA) based on Bray–Curtis dissimilarity (an abundance-based beta diversity metric) showing the overall beta diversity structure of microbial communities across all samples. Each point represents an individual sample. AD, orbital adipose tissue; CS, conjunctival sac swab; NS, deep nasal swab; ST, stool; SW, sterile water negative control; T–EN, tube–environmental controls. (**c**) Genus-level relative abundance profiles of individual samples, illustrating overall community composition and inter-sample variability. (**d**) Mean relative abundance of dominant genera summarized at the group level. (**e**) Heatmap of genus-level microbial abundance patterns across sample groups. Relative abundances were Z-score–normalized (mean-centered and scaled to unit variance) within each genus to highlight group-associated enrichment trends. ANPR represents the Allorhizobium–Neorhizobium–Pararhizobium–Rhizobium complex.

Following validation of the scRNA-seq dataset, we next assessed the structure and reliability of the microbiome data. Principal coordinate analysis (PCoA) based on Bray–Curtis dissimilarity revealed distinct clustering patterns across different sample types, indicating structured microbial community variation. Genus-level relative abundance profiles demonstrated consistent community composition across samples while capturing inter-sample variability. Alpha diversity analysis showed that sterile water controls exhibited markedly lower diversity compared with biological samples, supporting effective discrimination between background contamination and putative tissue-associated microbial DNA signals. Heatmap visualization of genus-level abundance patterns further highlighted group-associated microbial signatures, supporting the robustness of the dataset and the effectiveness of contamination control strategies^34^ (Fig. 6).

### Microbiome contamination assessment

Given the inherently low bacterial biomass of OA, technical validation was performed to evaluate and control potential environmental and reagent-derived contamination in 16S rRNA gene sequencing data, following established principles for low-biomass microbiome studies^35^. Environmental negative controls, including sterile water and blank samples, were processed in parallel with biological samples across DNA extraction, library preparation, and sequencing to monitor background signals.

All samples were sequenced targeting the bacterial 16S rRNA V3–V4 region using Illumina platforms. Across two sequencing batches, more than 11 million raw reads were generated. Only samples meeting predefined sequencing depth and quality thresholds were retained, with qualified OA samples typically yielding approximately 10⁴–10⁵ reads per sample, sufficient for downstream microbial community analysis.

Sequence data were processed using the QIIME 2 (version 2025.10) pipeline with DADA2 for denoising, quality filtering, and chimera removal, resulting in a high-confidence amplicon sequence variant (ASV) table. To assess contamination, microbial profiles of OA samples were compared with those of negative controls using alpha diversity metrics and Bray–Curtis-based beta diversity analyses. Principal Coordinate Analysis demonstrated clear separation between OA samples and negative controls, indicating that tissue-associated microbial profiles were distinct from background contamination.

Taxa consistently enriched in negative controls were identified as potential contaminants and removed using statistical decontamination approaches. After contaminant filtering, the retained dataset was considered to enrich for putative tissue-associated microbial signatures while mitigating background noise. Collectively, these validation procedures support the robustness of the sequencing data and the reliability of downstream microbiome analyses.

Given the inherently low microbial biomass of orbital adipose tissue, computational quality control was essential to isolate putative tissue-associated microbial DNA signals from environmental noise. Aseptic sampling procedures were paired with an extensive suite of negative controls, including sterile buffers, sampling swabs, and surgical instruments. To systematically partition endogenous microbiota from exogenous artifacts, we employed a dual-algorithm decontamination strategy. Initially, the Bayesian framework SourceTracker (v0.9.1) was applied to quantify the proportion of environmentally derived sequences within the orbital samples^36^. Subsequently, we utilized the *decontam* R package (v1.31.0) to statistically prune spurious taxa based on their prevalence profiles in the negative controls^37^. We evaluated a gradient of probability thresholds (ranging from 0.01 to 0.9). Crucially, even under the most conservative stringency parameter (0.01), different bacterial signatures remained stable, supporting their consistency with a tissue-associated origin, though viability cannot be confirmed by amplicon sequencing alone rather than being procedural artifacts. Furthermore, cross-referencing these orbital profiles with diverse anatomical sites, including fecal, deep nasal, and conjunctival samples, enabled the precise categorization of taxa into locally resident, gut-translocated, or contaminant populations, thereby enhancing the reliability and interpretability of the dataset.

## Usage Notes

Researchers should exercise caution when processing microbiome data. We recommend utilizing the environmental control data provided when reanalyzing raw 16S rRNA reads to establish custom filtering thresholds and mitigate batch effects. Regarding the single-cell RNA sequencing data, the provided object includes annotations for major cell types. However, sub-clustering of fibroblasts and lymphocytes, is highly encouraged to fully explore different cellular states. Finally, researchers analyzing the dataset should account for the cross-sectional nature of the cohort. Analytical models should be designed to evaluate correlative associations rather than drawing definitive causal conclusions regarding disease initiation or tissue remodeling.

The key cell-state observations reported in this study require independent experimental validation through orthogonal approaches, for example quantitative RT-PCR, immunohistochemistry, or flow cytometry, conducted in larger and independent surgical cohorts. The present data descriptor is centered on data generation and technical quality control. Future investigations employing these complementary methodologies represent a necessary step toward translating the present transcriptomic findings into an improved biological understanding of TED pathogenesis.

Several limitations govern the interpretation of the provided dataset. Orbital adipose tissue harbors a low microbial biomass, and while computational decontamination protocols were applied. 16S rRNA gene sequencing detects nucleic acids rather than viable microorganisms and does not differentiate among DNA originating from viable bacteria, non-viable bacteria, or circulating cell-free DNA fragments. Accordingly, terms such as microbiome, microbial community, and colonization as used throughout the dataset refer to DNA-based taxonomic profiles and should not be interpreted as evidence of active bacterial colonization. Demonstration of microbial viability within orbital tissues will require complementary approaches, including microbial culture, metatranscriptomic profiling.

Furthermore, tissue dissociation required for single-cell RNA sequencing disrupts the native architectural integrity of the orbital microenvironment. Cellular interactions, particularly the localized crosstalk among infiltrating immune populations, resident fibroblasts, and putative microbial antigens, occur within organized spatial domains. The absence of spatial transcriptomic mapping prevents the precise anatomical localization of specific cellular niches driving disease pathogenesis. Additionally, the observational study design captures a singular temporal snapshot of a dynamic autoimmune process. Thyroid-associated ophthalmopathy progresses through distinct clinical phases, transitioning from acute inflammation to chronic fibrosis. The provided dataset cannot resolve whether the observed transcriptomic shifts and microbial alterations initiate the disease cascade or represent secondary responses to orbital tissue remodeling. Longitudinal sampling is required to establish causal trajectories. Finally, amplicon sequencing targeting the V3 and V4 hypervariable regions provides restricted taxonomic resolution, typically failing to identify microbes at the species or strain level. Amplicon data provides no direct evidence of microbial metabolic activity or functional capacity, meaning transitioning toward shotgun metagenomics will be essential to elucidate the precise biochemical pathways mediating interactions between the host and microbes in the orbit.

In addition, the cell-state observations reported in our dataset would benefit from independent experimental validation using orthogonal methods such as quantitative RT-PCR, immunohistochemistry, or flow cytometry in independent surgical cohorts. We explicitly encourage and look forward to these efforts as the next logical step toward translating these transcriptomic findings into a deeper biological understanding of TED pathogenesis. We note that the clinical landscape for TED has shifted substantially since sample collection: the approval of teprotumumab N01^38^ / IBI311^39^ in China (March 2025) has markedly reduced the availability of orbital decompression specimens, and post-treatment tissues may not be directly comparable to pre-treatment samples. The present dataset therefore represents a pre-biologic-treatment reference for future validation studies.

## Data Availability

All raw and processed datasets generated or used in this study are available in public repositories. Raw single-cell RNA sequencing data have been deposited in the Genome Sequence Archive for Human (GSA-Human), China National Center for Bioinformation, under Bioproject PRJCA067648^40^ accession HRA019310^27^. Processed single-cell gene–cell count matrices, barcode files, and feature annotations are available in Zenodo at https://doi.org/10.5281/zenodo.18983916^28^. Raw 16S rRNA gene sequencing data are available in the NCBI Sequence Read Archive under SRA Study accessions SRP682191^29^ and SRP682192^30^. Previously reported orbital adipose tissue 16S rRNA data are available under SRP341968^31^. Processed microbiome feature tables and taxonomic annotations are available in Zenodo at https://doi.org/10.5281/zenodo.18385494^32^ and https://doi.org/10.5281/zenodo.18983916^28^.

## Code availability

No custom algorithms were developed for the present study. All data processing and analyses were conducted using established, open-source software packages that are adopted in the biomedical and bioinformatics research communities, ensuring transparency and reproducibility.

Single-cell transcriptomic data were processed using Cell Ranger (v10.0.0) with the GRCh38 human reference genome, following the manufacturer’s standard pipelines for read alignment, barcode processing, and gene expression quantification. Downstream single-cell data analyses, including quality control, normalization, dimensionality reduction, clustering, marker-gene identification, and differential expression analysis, were performed using Seurat (v5.3.1). Putative doublets were identified and removed using scDblFinder (v2.0.4).

Microbiome data analyses were carried out using QIIME 2 (version 2025.10), with denoising and amplicon sequence variant inference performed using DADA2. Taxonomic assignment was conducted using the SILVA database. Identification and removal of potential contaminant features were performed using the decontam package from Bioconductor and SourceTracker (v0.9.1), employing established statistical methods for contaminant detection.

All software tools used in this study are publicly available and can be accessed at the following resources: Cell Ranger (v10.0.0) (https://support.10xgenomics.com/single-cell-gene-expression/software/pipelines/latest/what-is-cell-ranger), QIIME 2 (version 2025.10) (https://qiime2.org/), DADA2 (https://benjjneb.github.io/dada2/), SILVA (https://www.arb-silva.de/), Seurat (v5.3.1) (https://satijalab.org/seurat/), scDblFinder (v2.0.4) (https://bioconductor.org/packages/release/bioc/html/scDblFinder.html), Decontam (https://bioconductor.org/packages/release/bioc/html/decontam.html), SourceTracker (https://github.com/danknights/sourcetracker).
